# Explainable AI-enabled hybrid deep learning architecture for breast cancer detection

**DOI:** 10.3389/fimmu.2025.1658741

**Published:** 2025-08-27

**Authors:** Yan Zou, Puyang Miao

**Affiliations:** ^1^ College of Mathematics and Computer Science, Chifeng University, Chifeng, China; ^2^ General Surgery Thoracic Surgery, Chifeng Songshan Hospital, Chifeng, China

**Keywords:** breast cancer, ultrasound analysis, XAI, deep learning fusion, interpretable medical imaging, VGG16, DenseNet121, Xception

## Abstract

**Introduction:**

Breast cancer stands is a leading prevalent and potential fatal infection affecting women worldwide, posing the requirement of a reliable and interpretable diagnostic system. The Deep Learning (DL) methods highly contribute towards medical imagery analysis but due to the black-box nature, its clinical adoption is limited due to lack of interpretability.

**Methods:**

This proposed work introduces a hybrid Deep Learning (DL) framework for that integrates three distinct convolutional neural network (CNN) pre-trained architectures: DENSENET121, Xception and VGG16. The proposed fusion strategy enhances feature representation and classification performance through model integration. To address the DL's black-box nature and promote clinical acceptance, the proposed framework incorporates an explainable artificial intelligence (XAI) component utilizing GradCAM++.

**Results:**

Experimental evaluation on benchmark breast cancer datasets demonstrates improved classification accuracy by approximately 13\% compared to individual models, demonstrating high performance of the fusion method with an accuracy of 97\%.

**Discussion:**

The use of fused DL model enhances the performance of the classification system offering higher accuracy and robust feature extraction. With the introduction of XAI, the cancer classification system presents interpretable results making it applicable in clinical contexts. GRADCAM++ method highlights the multiple lesions with finer edges from the ultrasound images that leads towards the model’s predictions, offering transparency and aiding medical professionals in diagnostic validation.

## 1 Introduction

Breast Cancer (BC) continuous to be most common and fatal malignancy impacting numerous female citizens worldwide. According to a study in (1), 31% cancer cases across the world are of Breast Cancer. Early detection of BC is essential to increase the survival rates of patients and offer improved quality of life (2). The conventional diagnostic techniques of BC, like mammography, ultrasound examination, and histopathological analysis, face difficulties like time-consuming process, human errors, and inter-observer variability that lead to high false-positive rates of BC detection (3). Early detection of BC is still critical, despite tremendous improvements in the diagnostic methods, primarily due to variable tumor morphology and different imaging artifacts (4). BC has evolved as an emerging area of research among the research community due to its significant social and economic impacts. More reliable, precise, accurate, and non-invasive techniques are needed for early identification of BC (5).

Medical Imaging and healthcare diagnostic methods have witnessed a huge transformation in recent years, mainly due to Artificial Intelligence (AI), especially the DL and ML architectures (6). These techniques have shown tremendous improvements in BC detection through various modalities like Ultrasound analysis, histopathology images, and mammogram analysis. These data-driven techniques facilitate the study of the massive volume of imaging and non-imaging data and capture deep, complex patterns that help to identify the tumor detection, which is often missed by human observers (7) (8). The recurrent networks and the CNN models have demonstrated higher accuracy in extracting features from large datasets and detecting malignancies (9). The scalability and reliability of such models make them ideal for analyzing medical imagery and help medical professionals to reach an informed decision about malignancy.

Single DL model faces difficulties in capturing complex and hidden patterns from medical imagery data, resulting in poor classification accuracy (7). The integration of multiple DL to overcome the limitations of individual models is an up-andcoming solution (10). Fusing different DL models at intermediate layers and then enabling combined training to predict the outcome facilitates recognizing complex patterns, enhanced feature extraction and representation, and improved accuracy (11).Integration of different models allows us to leverage their strengths and facilitate enhanced feature extraction resulting in improved accuracy. The fused models present a more generalized and reliable BC detection. Fusing different DL models is advantages compared to other ensemble techniques like voting and stacking. The voting and stacking methods aggregate the models output and present final prediction. These techniques do not indulge in retrieving complex patterns from the data which can be facilitated by Model Fusion. Both the voting learning and stacking perform at the output level whereas model fusion performs at intermediate layers and finds rich set of features by different models. All these features are combined and the final outcome is based on all these features. Thus model fusion results in improved performance compared to other techniques like voting and stacking.

Model Fusion refers to combining different models by take the advantage of strengths of various models to improve performance (12). Integration of different DL models is facilitated in three ways: Early Fusion, Intermediate Fusion and Late Fusion (13). Early Fusion focuses on integrating multiple data sources and then training a single model for analyzing the combined data (14). The Intermediate fusion refers to the extraction of features from data by different DL models. These extracted features by different models are then concatenated and a combined training takes place, resulting into final outcome (15). The Late fusion combines the decisions of individual models when implemented on the data. The majority outcome value presented by models is selected as the final prediction in Late Fusion (16). The proposed methodology implements the Intermediate Layer Fusion technique to integrate the strengths of VGG16, DenseNet121 and Xception models. These models extract the partial features from data which are then fused and after combined training and passing through fully connected layer, the final prediction (outcome) is obtained.

The major drawback that hinders using DL models for Breast Cancer Detection is their black-box nature (17). The DL models present the resultant outcome to the user and do not present specific explanations regarding how the outcome is derived. The medical professionals need not be satisfied with only the model’s outcome; they also need to know the insights behind the model’s predictions (18). The domain of Explainable AI (XAI) is gaining popularity as it overcomes the explainability limitation of DL models. These explanations help develop trust among the medical professionals and clinicians regarding the model’s prediction for Breast Cancer (19). The novelty of this study is to employ model fusion that integrates the strengths of three different DL models and the results are interpreted by GRADCAM++ to ensure the feeling of trust among the clinicians. The GRADCAM++ technique generates the pixel heatmaps that result in higher explainability for visual data. Whereas the models like SHAP and LIME are model-agonistic and show lower performances for high dimensional data. GRADCAM++ results in better localization by denoting the importance of each pixel through use of gradient computation at second level. Majority approaches for Breast Cancer Detection employ single modality approach or focus on ensemble techniques and lack explainability. Our proposed methodology fulfills this gap by implementing model fusion approach with interpretability using XAI technique.

**Figure 1:**
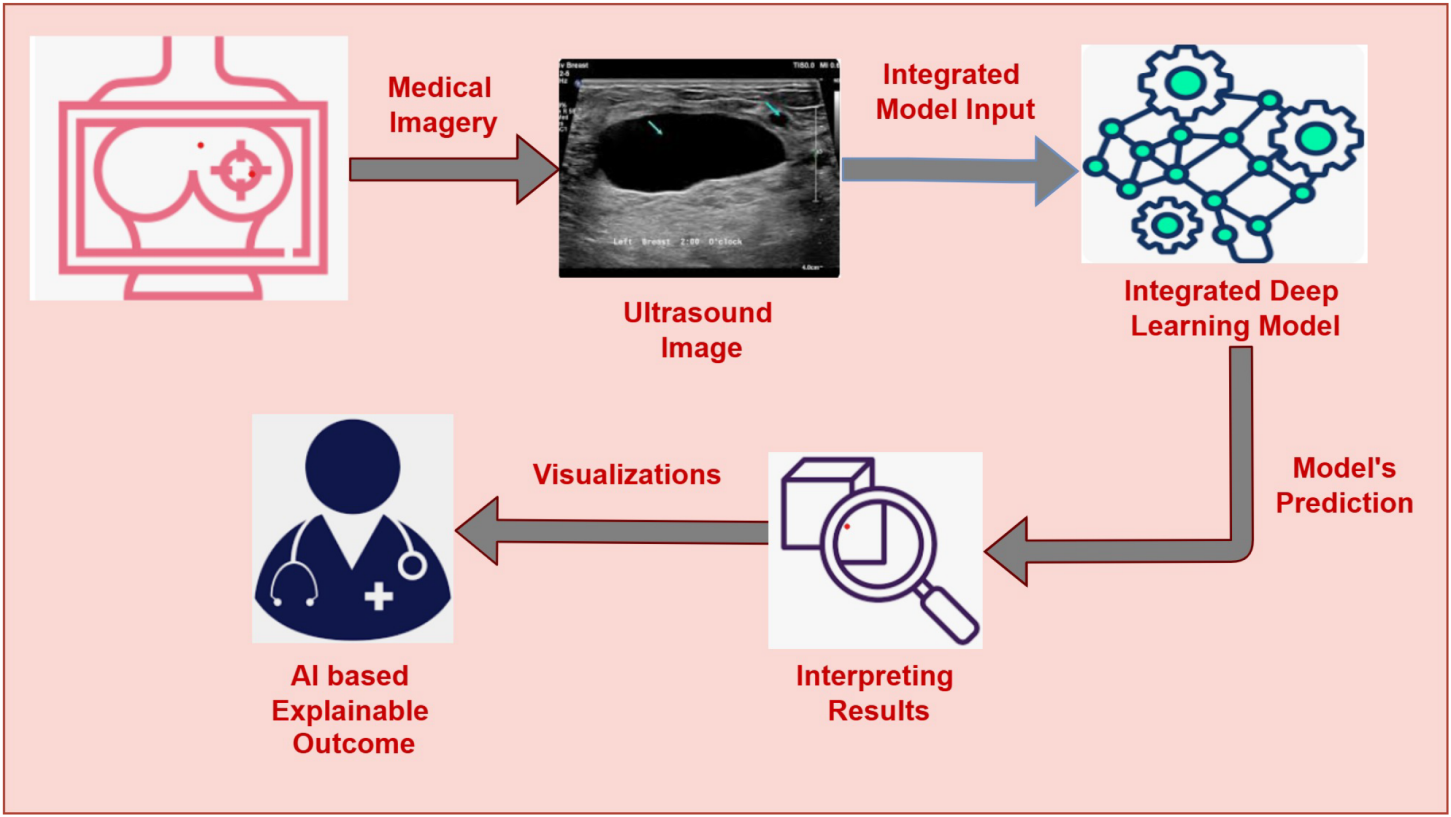
AI-driven interpretable breast cancer detection system.

The primary motivation of our work is to develop an AI-assisted, explainable Breast Cancer Detection system that enables early-stage detection of BC and helps to increase survival rates. The main motivation of this work is to assist the clinicians and radiologist in early and reliable breast cancer detection. Many AI systems for BC are insufficient to capture complex patterns and struggle to provide interpretations, which hinders clinicians from adapting them to their regular practice. Moreover, the varying size of cancer cells, texture, and shading effects in the breast’s ultrasound images impose challenges for most of the existing AI-assisted cancer detection systems. This study proposes the fusion of three CNN-based architectures, namely VGG16, DenseNet121, and Xception, leveraging their strengths to fetch deep features from the ultrasound images and enhance accuracy. The decisions presented by the model are interpreted by Gradient-weighted Class Activation Mapping ++ (GRADCAM++) (20). GRADCAM++ is the advanced version of GRADCAM that presents robust visualizations by highlighting the specific regions within the image that helps the model to reach the decision. GRADCAM++ with the DL fusion model offers enhanced accuracy, interpretable outcomes, and well-informed decision-making. This AI-assisted Interpretable BC detection system gives clinicians a well-suited data-driven prediction and helps them validate the predictions through visual representations. Our research attempts to develop a robust, interpretable, and reliable AI-driven diagnostic framework for BC detection, as depicted in **Figure 1**. Our proposed approach facilitates two important tasks: (i) Integration of three DL architectures (VGG16, DenseNet121, and Xception) for classifying images into benign or malignant with enhanced accuracy and (ii) Using GRADCAM++ to present insights about the predictions through image visualizations that show the specific sections inside the images which dominate the model’s precision. This work aims to bridge the gap AI-based diagnostic frameworks and traditional clinical practice by integrating a fused DL model with explainability through GRADCAM++, opening the door to a more dependable breast cancer detection system.

The major contributions of our research are listed below:

- Implementation of deep fusion learning framework integrating VGG16, DenseNet121, and Xception to classify BC from the ultrasound images.
- Facilitating explanations of the fused model’s outcome through the XAI technique-GRADCAM++ that presents the highlighted section among the image driving the model’s decision.
- Experimental evaluation on the Breast Ultrasound Image Dataset to demonstrate the performance of the fused model and assess its performance with different state-of-the-art techniques.

The structure of this research paper is as follows: The state-of-the-art methods employed for BC detection are reviewed in Section 2. Our proposed methodology, with the algorithm stating minute relevant details, is demonstrated in Section 3. Dataset’s Description and the experimental parameters used for the study are explained in Section 4. Experimental results and their comparison with the state-of-the-art methods are shown in Section 5. Finally, the paper presents the Conclusion along with the Future Scope in last Section 6.

## 2 Related work

Over the years, researchers have put forth numerous approaches to identify Breast Cancer (21, 22). A systematic study of existing literature is carried out to gain a thorough grasp of the recent developments in the relevant domain. Keyword-based search was first performed utilizing phrases like AI for early BC detection, DL in cancer diagnosis, ultrasound image analysis, breast cancer detection, and mammography image processing. IEEE Xplore, Elsevier, SpringerLink, Scopus, Google Scholar, and other well-known databases were searched using these keywords. Priority was given to studies focusing on minimally invasive or non-invasive diagnostic methods. Research using machine learning or deep learning models, particularly those with explainable results, was considered in the literature. In addition to highlighting the key developments in breast cancer detection research, this systematic review technique identifies essential gaps and obstacles that prevent the broad use of AI-driven diagnostic systems in clinical practice. The clinical knowledge and patient assessment by radiologists and pathologists have historically been crucial in diagnosing breast cancer (23). Nowadays, advancements in Artificial Intelligence (AI) have revolutionized medical imagery, providing robust image analysis. In recent years, there has been much promise for AI-based systems to help interpret ultrasound images and mammograms (24). The use of Machine Learning is trending for extracting handcrafted features. The machine learning models face difficulties in capturing the complex intrinsic features, which may restrict their generalizability (25). However, the development of deep learning methods, which are driven by increasing computing power and due to availability of massive datasets, has significantly enhanced performance in the automatic detection of breast cancer (26). The CNN model and its various variants are effective for medical image analysis. The deep neural networks face the issue of interpretability due to their black-box nature, which hinders their acceptance in clinical settings (27).

The researchers in (28) presented the use of five fine-tuned pre-trained models (Xception, InceptionV3, VGG16, MobileNet, RESNET50) on a GAN-augmented Image dataset with multiple magnifications. These models classify the Breast Magnetic Resonance Image (MRI) images into eight different categories (four benign and four malignant). The researchers in (29) demonstrate using the CNN pretrained model Xception on the MRI and the computed tomography (CT) images. This model is implemented on the public dataset from Kaggle and achieves high merit results in bifurcating between malignant and benign classes. In (30), the CNN architecture is implemented for analyzing thermal images to predict BC. This model is lightweight and faster than pre-trained models like RESNET50 and Inception. This model analyzes the augmented thermal photos to make a bigger dataset and records higher accuracy than non-augmented data. Similarly, the use of the CNN architecture for identifying BC is shown in (31). The RGB images are analyzed through 3 convolutional layers and three pooling layers, following a fully connected layer. The study in (32) presents an upgraded Deep CNN model that identifies four distinct abnormalities, moving beyond the standard benign/malignant binary classification. The proposed model achieves 88% classification accuracy by combining transfer learning with RESNET50 and proposing a novel adaptive learning rate technique that adapts dynamically based on error curves. This approach has tremendous potential to reduce radiologists’ diagnostic errors, particularly in detecting tiny characteristics, contributing to high false-positive rates in conventional screening. Jahangeer et al. in (33) implemented the VGG16 model for analyzing the mammography images. The images are preprocessed by a median filter that removes the noise and are then fed to the VGG16 model, and the deep abnormalities are extracted. The authors in (34) propose an efficient diagnostic pipeline that combines VGG16 feature extraction with machine learning classifiers to address computational constraints in histopathology image interpretation. This technique outperforms standard CNNs in binary (malignant/benign) classification while dramatically decreasing computational overhead. The strategic use of pre-trained VGG16 for high-level feature extraction avoids costly full-model training. The researchers in (35) present the use of the CNN model with the attention based mechanism that results in enhanced segmentation of ultrasound images. This model covers the local and global features leading to improved image segmentation. The authors in (36) introduce the use of the two tiered q-rung ortho-pair fuzzy sets for personalized breast cancer detection. This model results in expert consensus, improving reliability in clinical settings. The researchers in (37) presented the use of the tissue impedance management and analyzing them with LSTM model to predict early stage cancer. This model record higher accuracy and are suitable for real time applications. The study in (38) presents a novel architecture derived from systematic optimization of VGG16 via hyperparameter tuning and layer restructuring. The mammography images are augmented and preprocessed, where unwanted regions are removed, and then analyzed by the VGG16 architecture.

The study in (39) presents a hybrid DL method for BC diagnosis that combines bio-inspired Multi-Layer Perceptron (MLP) models and deep CNNs (GoogleNet and AlexNet) with different preprocessing methods. GoogleNet outperformed other techniques and records superior accuracy. The work demonstrates the complementary utility of optimization-based MLP models using statistical features and highlights the efficacy of pretrained CNNs for feature extraction. This dual strategy covers high-level feature learning and conventional feature-based categorization in mammography analysis. The researchers in (40) present a ensemble meta-learning technique that combines meta-optimization, data augmentation, and transfer learning (Inception, ResNet50, DenseNet121) to classify BC using. The hybrid approach overcomes the shortcomings of traditional deep learning in managing intricate lesion patterns through feature concatenation. The ensemble model’s preliminary results show better accuracy than standalone models. In (41), the authors present the use of transfer learning with models AlexNet, RESNET101, and InceptionV3 on an ultrasound image dataset. This approach uses the soft voting technique to present the final forecast based on the outcome probabilities of particular individual models. The voting learning model achieved higher accuracy compared to the individual models. Although the study focuses on improving model generalization, dataset size remains a constraint. The researchers in (42) introduce a transfer-learning approach for BC detection using breast cytology images, addressing critical diagnostic challenges in resource-constrained regions. This framework combines feature extraction from three pre-trained CNNs (GoogleNet, VGGNet, ResNet) with an average pooling classifier, demonstrating superior performance over isolated deep learning models. Experimental results on benchmark datasets reveal the system’s exceptional accuracy in classifying malignant vs. benign cells, outperforming conventional architectures through its knowledge-transfer mechanism. The work highlights how transfer learning can overcome data scarcity issues common in developing nations while improving diagnostic reliability. In (43), the authors address the significant difficulty of breast cancer classification in ultrasound imaging by creating an optimal VGG16-based transfer learning method. The proposed system has three components: median filtering for effective speckle noise reduction, VGG16 convolutional layers for feature extraction, and a unique two-layer Deep Neural Network (DNN) classifier with dropout regularization. The addition of Grad-CAM imaging allows for clinically interpretable localization of malignant characteristics, confirming the model’s concentration on diagnostically significant regions.

The researchers in (44) demonstrate the use of the multi-modal strategy that combines U-Net transfer learning model for image analysis and ensemble Random Forest (RF), CNN and Support Vector Machine (SVM) model for numerical feature processing and explainable AI (XAI) for clinical interpretation. The study gives vital insights into feature importance using SHAP, indicating that hybrid feature spaces increase the malignancy diagnosis of small lesions. The survey in (45) shows a breast cancer AI system by creating an interpretable prediction framework that combines different boosting algorithms (LightGBM, CatBoost, and XGBoost) and uses LIME-based model explanations. After hyperparameter adjustment, LightGBM outperformed the other models and recorded the highest accuracy compared to the others. The use of LIME presents locallevel interpretations that help clinicians validate predictions. The researchers in (46) propose an ensemble learning model combining the forecasts of DenseNet201, VGG19, and EfficientNetB7. This approach uses the attention-based mechanism of the pre-trained CNN models, which helps to concentrate on the vital regions of the image for prediction. The GRADCAM technique is used for explainability, which generates class activation maps and presents the important sections in the image that define the model’s outcome. The authors in (47) propose a lightweight attention model named DALARESNET50. This model integrates lightweight attention techniques with RESNET50’s fourth layer, integrating the Fully Connected layers to boost feature discrimination. Dynamic Threshold Grad-CAM produces more precise visual explanations than traditional Grad-CAM, with adjustable heatmap thresholds matching pathologists’ diagnostic focus areas.

The existing literature demonstrates recent developments in Explainable AI (XAI), ensemble modeling, and deep learning for detection of breast cancer. In order to improve the feature representation and enhance accuracy in medical imaging, a variety of convolutional neural network (CNN) models have been proposed. The implementation of a single model for detecting BC faces some troubles in identifying tiny or subtle lesions, poses a high risk of false positives, and has limited generalizability across datasets with different imaging conditions. Techniques that involved multiple models like Ensemble Learning are less appropriate for real-time clinical application because they frequently require high processing resources and result in higher latency, even if they have shown promise in addressing some of these issues. Although XAI approaches like Grad-CAM and LIME are investigated to lessen the black-box nature of DL models, many of these approaches still provide a limited degree of interpretability, concentrating mostly on visual overlays without providing complete support for clinical reasoning. The current study fills these gaps by presenting a fusion-based model that combines VGG16, Xception, and DenseNet121 to incorporate their complementing abilities to extract both fine-grained and high-level characteristics from breast ultrasound images. The evaluation is based on the Breast Ultrasound Image (BUSI) dataset (48), that offers a varied and clinically relevant testbed. Grad-CAM++ produces high-resolution, class-discriminative visual explanations for model interpretability, improving transparency and building clinician trust. The proposed method aims to compromise performance, interpretability, and clinical applicability in the context of breast cancer diagnosis by emphasizing effective multi-architecture fusion and incorporating cutting-edge XAI.

## 3 Proposed methodology

**Figure 2:**
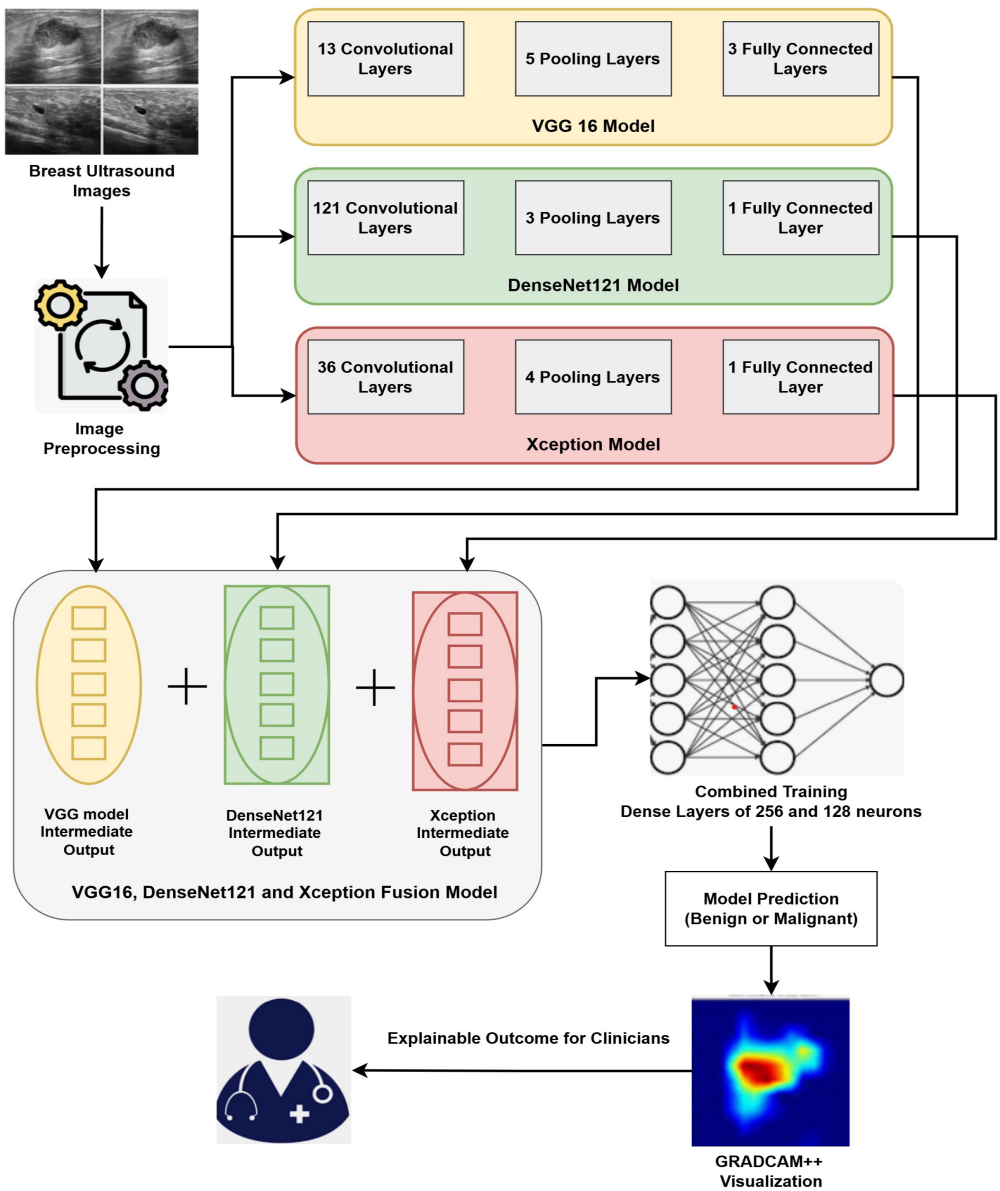
Hybrid Interpretable Fusion Model Architecture.

Our proposed methodology primarily accomplishes two goals: (i) Fusion of three DL architectures and (ii) Implementation of the XAI technique for explaining the results. Our proposed approach fuses three DL architectures, namely VGG16, DenseNet121, and Xception. Fusing these three modalities combines the strengths of the individual modality and facilitates extracting deep features from the Image data. The outcomes of the proposed fused model are then interpreted by GRADCAM++, which presents visualizations explaining the model’s decision. The detailed architecture of the fusion model is depicted in **Figure 2**.

The input images are pre-processed and are then applied to the hybrid model. In pre-processing, the input images are resized to 128*128 pixels to match the standard dimensions of all the three models. The pixel values of these images are then normalized and each pixel value is represented in the range of 0 to 1. The input images are augmented by applying slight rotation and flipping in order to avoid over-fitting and introduce generalizability. These resized, normalized and augmented images are then fed to the hybrid deep learning model. The hybrid model merges 3 pre-trained models: VGG16, DenseNet121, and Xception. Since each of these models contributes exceptional capabilities to the group, it facilitates extraction of various complementary patterns from breast ultrasound images. The VGG16 model is renowned for using tiny 3x3 convolutional filters and having a consistent design (49). Its three completely linked layers after 13 convolutional layers provide detailed spatial feature extraction, making it especially suitable for fetching complex patterns in medical imaging (50). Through dense connected layers, where each receiving inputs from previous layers, DenseNet121 architecture improves feature propagation (51). This design promotes feature reuse and lessens the vanishing gradient issue, both advantageous for medical picture classification applications. Xception’s simplified design, which is based on depthwise separable convolutions, effectively collects channel-wise and spatial information (52). It is appropriate for high-resolution medical images segmentation and analysis as it is excellent at learning discriminative features while lowering computing costs. Our fusion model extracts the output from the penultimate layer of each separate model and applies feature-level integration. A single, high-dimensional feature vector that captures multi-scale and multi-perspective data is created by concatenating these high-level features. The combined feature vector is subsequently run through a series of dense layers that are fully coupled to learn joint representations. Final classification is performed by an output layer using a sigmoid function as activation unit that is appropriate for the classifying binary outcomes like identifying benign versus malignant instances.

We use Grad-CAM++, a visualization method that offers fine-grained and class-discriminative saliency maps, to improve the interpretability of our model (53). Grad-CAM++ produces heatmaps that highlight the most critical areas in the input by computing gradients of the targets flowing into the last convolutional layer. Grad-CAM++ has superior localization accuracy, which is crucial in medical diagnostics, and is more adept at handling many instances of the same class in an image than conventional Grad-CAM. Grad-CAM++ creates a heatmap overlaying the original ultrasound image for every prediction, emphasizing the areas that most affected the model’s categorization. These visual representations shows the alignment of the model within the relevant areas of the images and help the clinicians to find the reasoning behind a particular prediction. Interpretable results can help validate decisions and boost confidence in AI-assisted technologies, which is why this type of interpretability is essential in delicate fields like breast cancer diagnoses. Combining a hybrid fused model with Grad-CAM++ explainability guarantees high predictive accuracy and clinical transparency, which makes the system ideal for use in actual diagnostic processes.

### 3.1 Proposed algorithm

This research demonstrates fusion-based DL framework that combines an explainable AI technique with multiple pretrained CNNs to improve the precision and interpretability of BC diagnosis. The proposed approach fuses the VGG16, DenseNet121, and Xception models at the intermediate layers, and then the combined training through multiple dense layers is performed. This training, through the fully connected layers, then facilitates cancer prediction at the output layer. The fused model maintains computational efficiency with frozen backbone networks while capturing a variety of spatial and contextual patterns by utilizing feature concatenation and joint training. Post prediction, the GRADCAM++ method presents interpretations by providing the highlighted sections in the Image that dominate the prediction by model. The steps and details of proposed work are explained in **Algorithm 1**.

**Algorithm 1:**
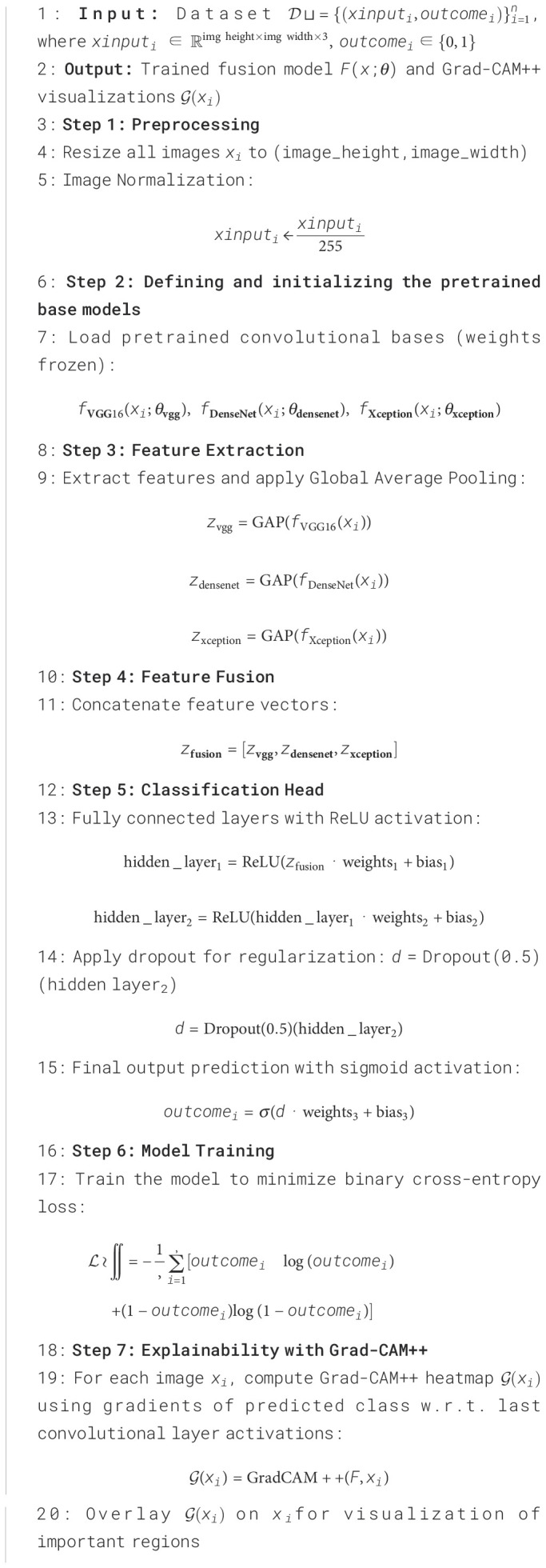
Fusion of VGG16, DenseNet121, and Xception with Combined Training and Grad-CAM++ for Breast Cancer Detection.

The working of **Algorithm 1** begins with the pre-processing task where the input images are resized to a size of 128 * 128 pixels, scaled to a consistent height and width, and the Image pixels are normalized in the range of [0,1]. This enhances training stability and guarantees uniformity in input dimensions. Post-preprocessing of Images, the base models (VGG16, DenseNet121, and Xception) are loaded and initialized. The three models that are fused together are pre-trained CNN models. These models are pre-trained on large dataset namely ImageNet. The weights of these pre-trained models are frozen to preserve the rich set of features retrieved from the large dataset. The pre-trained model weights are frozen to reduce over-fitting during the training phase on smaller datasets. With help of frozen weights, we extract stable feature representations that are then combined, encouraging consistent model fusion. The input images are then processed independently by each pretrained model, which extracts the relevant features. The features extracted by each model are then passed through Global Average Pooling (GAP) that reduces the spatial dimensions without sacrificing discriminative information. The GAP method averages the spatial locations and thus reduces over-fitting and results in less computational overhead during model training. GAP also helps to handle the variations that exists in the ultrasound images. The feature vectors extracted by different models are then concatenated to have enhanced feature maps that consist of various spatial and contextual patterns extracted by other architectures. The combined strengths of the three models—the depthwise separable convolutions of Xception, the dense connectivity of DenseNet121, and the hierarchical feature learning of VGG16—are combined into a single fusion vector. To enable non-linear feature transformation, the concatenated feature vector is processed through a classification head comprising two completely linked layers consisting of 256 and 128 neurons respectively, with ReLU activation functions. Dropout at each layer is used to prevent overfitting. The output layer uses a sigmoid function for activation to generate a binary prediction that indicates whether cancer is present. Grad-CAM++ creates heatmaps that highlight the specific regions in input images that impact the model’s outcomes in order to achieve interpretability. GRADCAM++ generates a visual explanation by calculating gradients of the anticipated class score concerning the activations of the final convolutional layer (54). The heatmaps generated by GRADCAM++ are mapped with the original image, and the final overlay representation is generated. This stage promotes trust and facilitates diagnosis by helping medical professionals validate the model’s outcomes.

### 3.2 Experimental setup

The performance of the proposed model is assessed through a set of varied experiments on the Breast Ultrasound Image dataset. All the experiments are performed on an Intel Core i7 laptop with a 2.8 GHz processing speed, 32GB DDR4 RAM, along with NVIDIA GeForce RTX 3090 GPU comprising of 24GB VRAM. The programming environment used for the experimentation is Anaconda Software with Python 3.as the programming language. Numerous libraries are required for performing experiments: Numpy, Pandas, Keras, TensorFlow, Matplotlib, OpenCV, and Sci-kit Learn. The Numpy library handles the mathematical computations. The Pandas library provides data handling functionalities, Keras and TensorFlow for implementing Deep Learning Models. The Matplotlib library is used for presenting visualizations and graphs. The OpenCV library is used for Image processing tasks like resizing images, normalizing and augmenting images. The Sci-kit learn library is used of evaluating the model with different evaluation metrics. The grad-cam library is used for implementing the GRADCAM++ technique for explaining the model’s decision.

The images are first resized to 128*128 pixels. To handle the class imbalance in the dataset, some photos are rotated to some extent and augmented to some scale. The resultant dataset is then normalized, where each pixel value is on the scale of [0,1]. The balanced normalized dataset is now divided into parts with ratio of 75:25. The first part comprising of 75% data is used for model training, and the left over 25% data is used for model testing. From the total training data, 10% data is used for model validation. Proposed Fusion model, consisting of three pre-trained CNN models (DENSENET121, Xception and VGG16), is fed with images with three channels. The fusion model is trained with for 50 epochs with the batch size of 32. We tested the experiments with batch sizes of 8, 16, 32, 64, and 128. The lower batch sizes of 8 and 16 resulted into slower gradient movements and higher batch sizes of 64 and 128 required higher computational resources like memory and resulted into higher gradient convergence. The batch size of 32 resulted into stable training of the model and balanced the trade-off between model convergence and computational requirements. Implementation of dropout regularization is used for avoiding model overfitting.

#### 3.2.1 Dataset description

The Ultrasound Breast Images for Breast Cancer dataset (48) is used for the experiments in our research. A large number of ultrasound images of breasts, divided into classes of benign and malignant tumors, make up the dataset. This dataset includes an extensive collection of ultrasound pictures of breast tissue that were gathered to help in breast cancer diagnosis. As a radiationfree, and non-invasive diagnostic technique, ultrasound imaging is frequently used to characterize breast lesions, especially when separating benign from malignant classes. A total of 8,116 ultrasound images in all, divided into two classes—malignant (4,042 images) and benign (4,074 images) are included in the dataset. Professional radiologists obtained and annotated the original pictures, guaranteeing clinical dependability and precise diagnosis representation. These annotations represent the expert-level knowledge needed for real-world diagnosis and act as the ground truth for supervised ML models. The images have a resolution of 224*224 pixels in JPEG format. Image Augmentation techniques like sharpening and rotation are applied to improve the dataset and handle possible issues with model generalization. By simulating genuine diagnostic settings and adding variability, these changes improve robustness and lower the chance of model overfitting. The summarized information about the dataset is presented in the **Table 1**.

**Table 1:** Summary of ultrasound breast images dataset.

Parameter	Value
Dataset Used	Ultrasound Breast Images for Breast Cancer
Imaging Modality	Ultrasound
Image Format	JPEG
Image Resolution	224*224 pixels
Count of Output classes	2 (Benign, Malignant)
Total Count of Images	8,116
Count of Benign Images	4,074
Count of Malignant Images	4,042
Data Augmentation Methods	Rotation, Sharpening
Annotation Source	Clinical Radiologists

#### 3.2.2 Evaluation metrics

Four standard classification metrics, namely Accuracy, Recall, Precision, and F1 Score, are used to evaluate the model’s efficiency (55). These metrics assess the capacity of model to accurately detect between malignant and benign BC cases. Out of all the predictions performed, the accuracy measure shows the percentage of correctly identified observations (25). When the distribution of classes is pretty balanced, it is beneficial as a general indicator of overall performance. Accuracy is helpful when the dataset is balanced, but when there is a class imbalance, as is typical in medical diagnostics, accuracy might not accurately reflect the model’s efficacy (56). Thus, we also consider the F1 score, recall, and precision,. The ratio of correctly predicted positive (Malignant) instances to all expected positives is precision. It highlights how the model may avoid false positives, essential for medical diagnosis to prevent needless worry or medication (56). The capacity of the model to detect all real positive (malignant) cases is measured by Recall, sometimes referred to as sensitivity (57). It is particularly crucial in medical settings because a false negative result could have significant repercussions if a malignant case is not detected (58). F1 Score is the recall’s and precision’s harmonic mean (57). It offers a single performance indicator that strikes a compromise between the two, making it especially useful in datasets with class imbalance.

## 4 Result analysis

The performance of the proposed DL fusion model in detecting BC from breast ultrasound pictures is assessed through various experiments. Using the publicly accessible ultrasound breast cancer image dataset, which includes both malignant and benign classifications, the model’s performance was carefully evaluated. Three popular CNNs—Xception, DenseNet121, and VGG16—are integrated in the proposed fusion model. By utilizing each architecture’s complementary characteristics, this fusion strategy tends to enhance the performance and resilience of breast cancer categorization. The experiments in this study were conducted in two phases. The first phase demonstrates the implementation of multiple deep learning architectures on the dataset, and the second phase reflects the fusion of the top three performing models in the first experiment. Later, GRADCAM++ is employed for Image Interpretation.

In the first phase, various standalone CNN-based architectures are implemented and the results of each model are noted. VGG16, AlexNet, RESNET50, GoogleNet, Inception, MobileNetV2, Xception, DenseNet121, and EfficientNetB0 are among the models considered, due to their wide use in medical Imagery Analysis. To accurately classify ultrasound data into malignant and benign classes, each model is adjusted and fine-tuned to learn pertinent features. Among the total data, 75% data constitutes the training data and left over 25% constitutes the testing data. This division provided enough data for training while guaranteeing a trustworthy assessment on unseen data. A batch size of 32 was used to train all models for 50 epochs, allowing for learning intricate patterns within a manageable computational cost.

The experimental findings showed that VGG16, DenseNet121, and Xception models with accuracies of 84%, 83% and 82% respectively, performed better than the other models. Their continuously high performance proves the robustness and dependability of these three models in identifying breast cancer. In the second phase, these three models are fused with the goal of improving classification performance even more. The Image dataset is partitioned according to the same 75:25 ratio, where 75% is training data and the remaining 25% is the testing data. The model is trained with Adam optimizer over a range of iterations (epochs), ranging from 10, 20, 35, 50, 70, to 100. The model observed a rise in validation losses after 50 epochs, and hence, the model training is performed for 50 epochs. By utilizing enhanced feature extraction capacities of all the three models, the fusion model shows higher classification accuracy. The accuracy achieved by fusion model is 97.14%. The performance results of various models are shown in **Table 2** below.

**Table 2:** Performance results of various architectures on breast ultrasound image dataset.

Architecture	Accuracy	Precision	Recall	F1 score
AlexNet	62.32%	62.32%	62.32%	62.32%
GoogleNet	76.43%	70.23%	78.34%	74.28%
RESNET50	70.8%	69.4%	71.5%	70.4%
MobileNetV2	71.1%	71.1%	71.1%	71.1%
EfficientNetB0	69.33%	69.55%	70.65%	70.1%
Inception	78.8%	77.41%	79.52%	78.45%
VGG16	84.43%	80.65%	90.40%	85.25%
DenseNet121	83.54%	84.36%	83.54%	83.45%
Xception	82.45%	83.15%	82.24%	82.37%
FUSION MODEL	97.14%	95.96%	98.42%	97.18%

**Figure 3:**
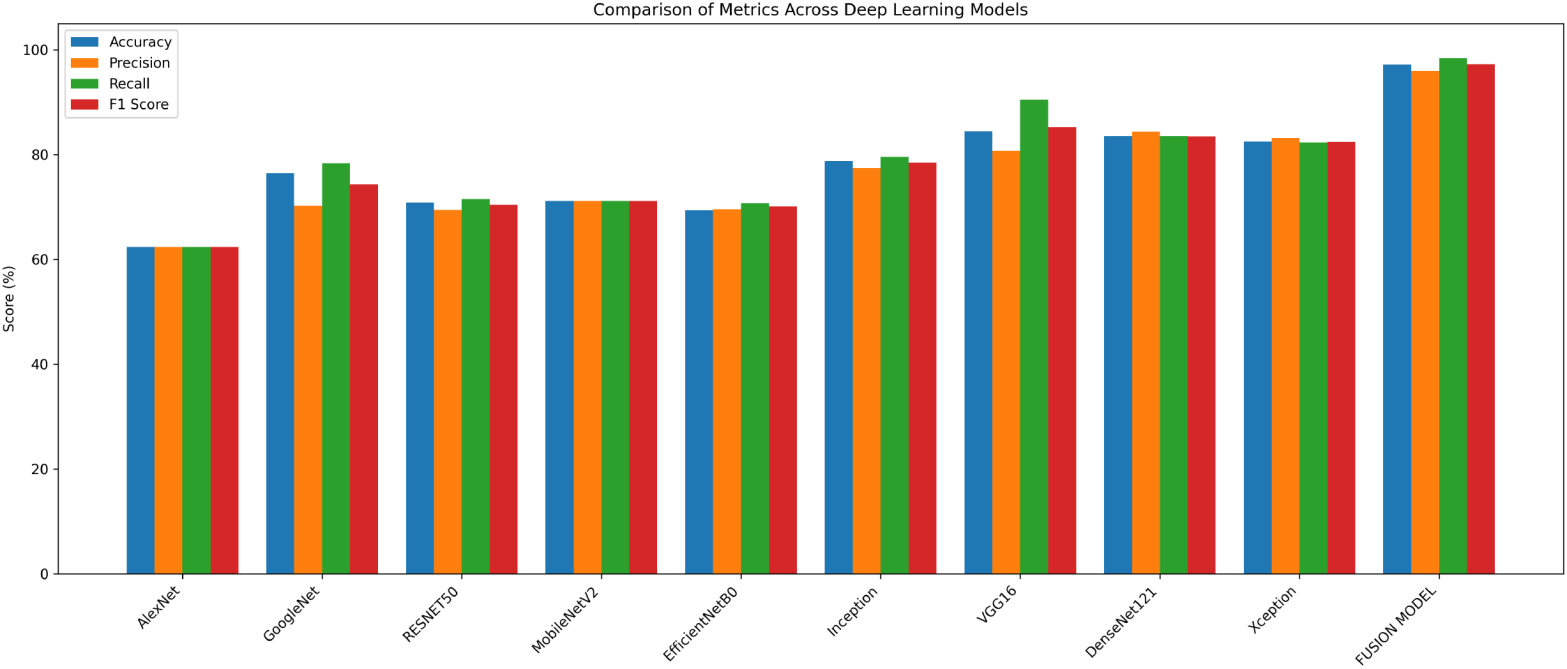
Comparative performance of different models for breast cancer detection.

The fusion model shows superior performance compared to the individual models and records 97% accuracy, which is around 13% higher than VGG16, the best-performing individual model. The comparison of performance of different models is presented in **Figure 3**.

**Figure 4:**
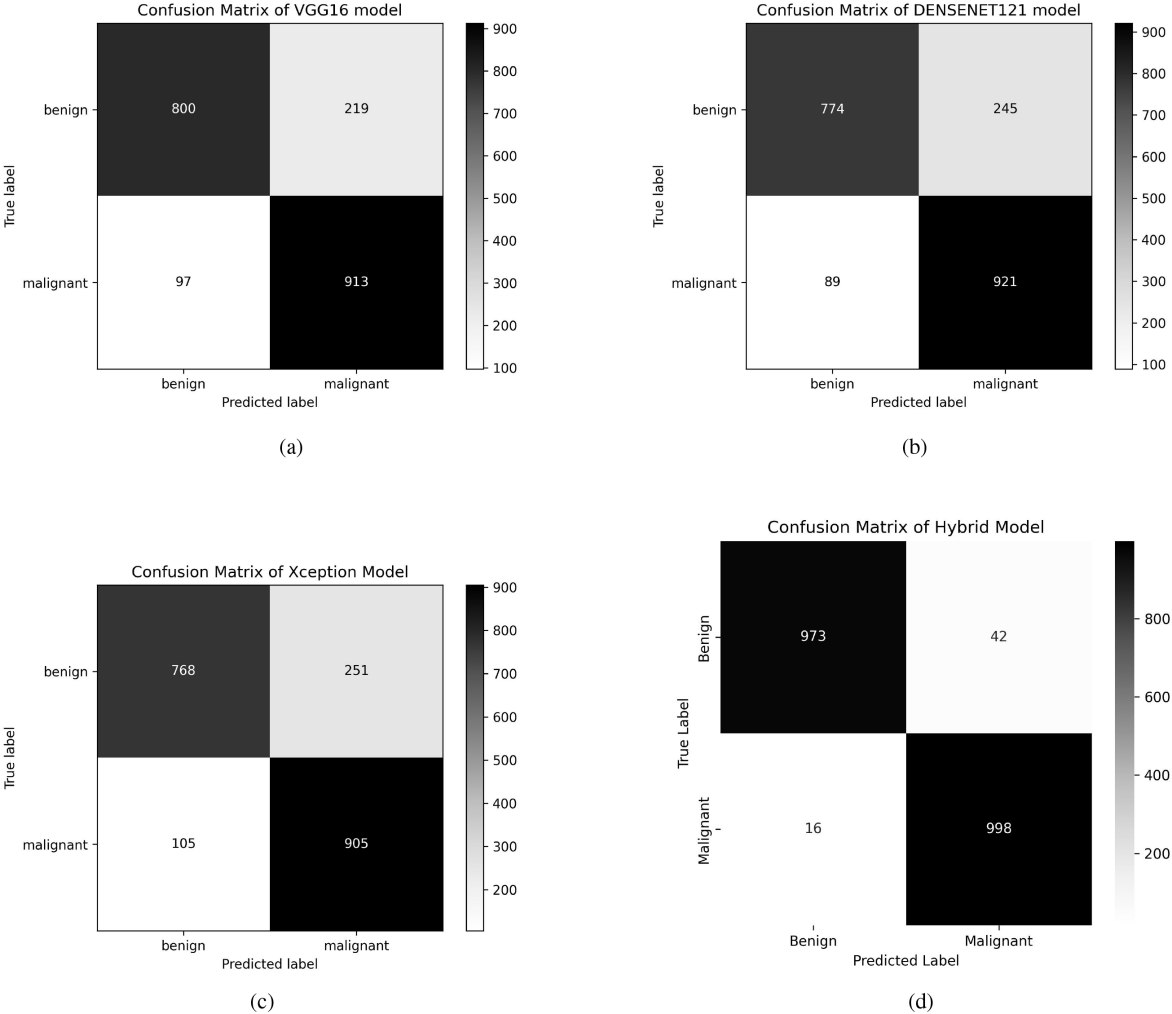
Confusion matrices of VGG16, DenseNet121, Xception, and Fusion models. **(a)** Confusion matrix of VGG16 model, **(b)** Confusion matrix of DenseNet121 model, **(c)** Confusion matrix of Xception model, **(d)** Confusion matrix of Fusion model.

This enhancement in performance is achieved by the robust feature extraction capacities of the individual models that assist one another throughout the fusion process. The confusion matrix of the individual models and the fused model is shown in **Figure 4**. By displaying the number of properly and incorrectly predicted data, the confusion matrices demonstrate how well each model performs in categorization. The fusion model demonstrates improved prediction performance and produces more accurate classifications by integrating the benefits of three distinct architectures.

The proposed methodology proposes using Grad-CAM++ to highlight the crucial regions in ultrasound images that affect classification choices to improve the interpretability of the fused model. Grad-CAM++ creates class-specific localization maps, allowing a more thorough comprehension of the model’s decisions. The regions the fused model considers most important for categorization are highlighted in the generated visuals. According to our study, the model primarily concentrates on biologically significant cellular structures linked to breast cancer, which supports the accuracy of its predictions and their congruence with domain knowledge.

**Figure 5:**
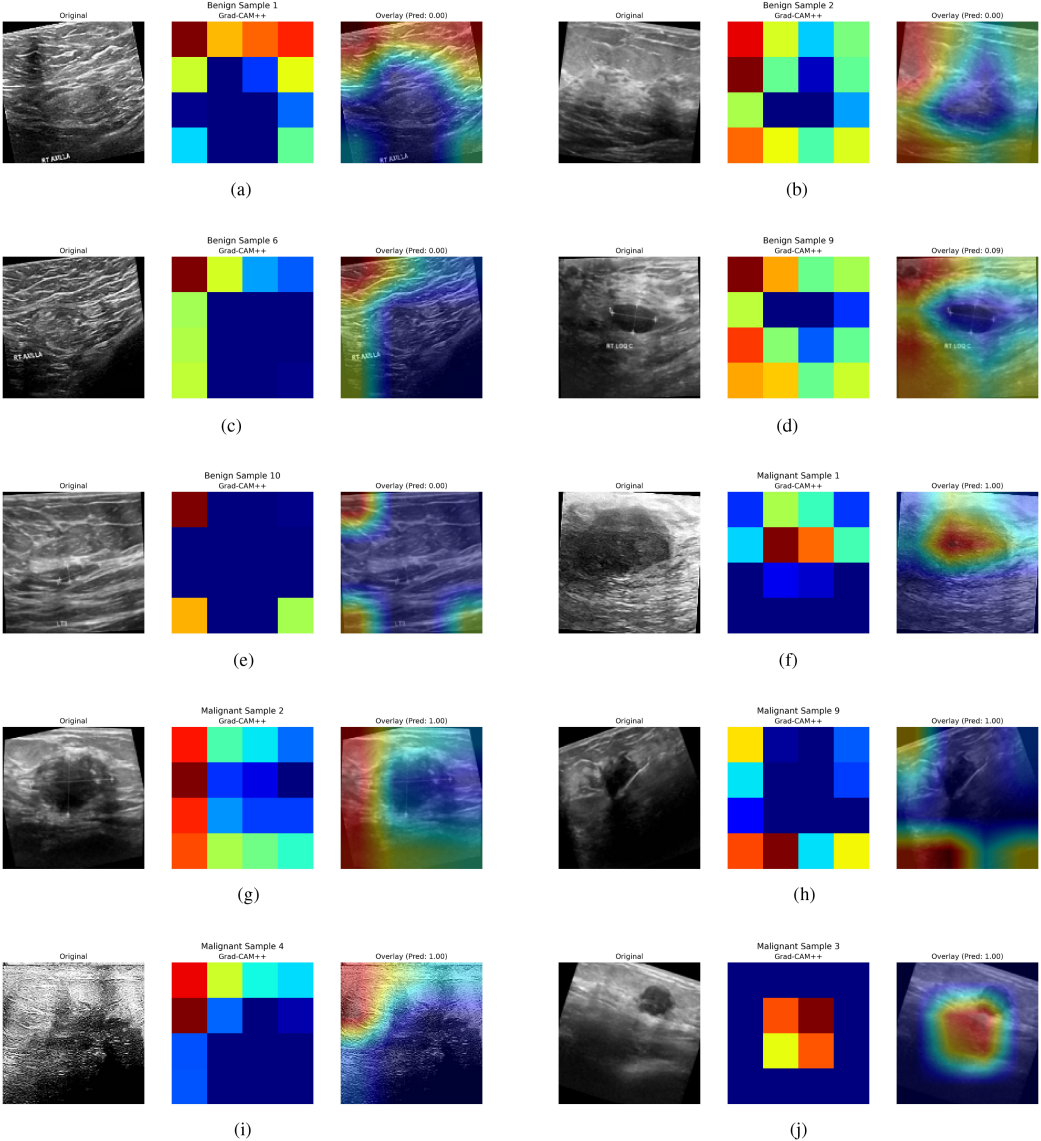
GRADCAM++ interpretations for Model Predictions. **(a)** Benign tumor example 1, **(b)** Benign tumor example 2, **(c)** Benign tumor example 3, **(d)** Benign tumor example 4, **(e)** Benign tumor example 5, **(f)** Malignat tumor example 1, **(g)** Malignat tumor example 2, **(h)** Malignat tumor example 3, **(i)** Malignat tumor example 4, **(j)** Malignat tumor example 5.

The results of GRADCAM++ for interpreting the model’s predictions are demonstrated in **Figure 5**. Every individual result comprises three different image sections. The first section comprises of the original breast Ultrasound Image, which is selected from the testing dataset. The second part of the image results is the GRADCAM++ heatmap. The spatial activation patterns displayed in this heatmap influence the classification output. High model attention is indicated by red and yellow, which show locations that significantly impact the prediction. On the other hand, areas that are in blue or black color indicate a minimal or insignificant impact. This stand-alone heatmap isolates the feature map regions given priority during inference, offering an abstract “model-centric” approach. The image’s third part is the original image’s overlay and the heatmap presented in the first and second parts. The GRADCAM++ heatmap is superimposed on the original image. The overlay uses a color scale, with blue/black areas denoting little attention and red/yellow areas denoting high attention. For example, Overlay (Pred: 0.00) suggests a confident benign diagnosis, whereas Overlay (Pred: 1.00) indicates a confident malignancy. The title includes an annotation on the prediction probability. With this visualization, clinicians can better relate model attention to clinical and anatomical aspects, including shadowing, irregular edges, and masses. As shown in **Figures 5a–e**, the GRADCAM++ heatmaps rarely consist of red color boxes denoting high chances of malignancies. The resultant overlay image demonstrates the Prediction of a 0.0 value, and the critical region is highlighted in blue. This helps the clinician recognize cases of benign tumors easily. Similarly, the **Figures 5f–j** demonstrate the cases of malignancies. These images show that the GRADCAM++ heatmaps consist of red color boxes defining the high-level attention of the model, demonstrating cases of malignancies. These heatmaps are then superimposed on the original images, generating the resultant overlay image. The prediction in this case is equal to 1.00, demonstrating malignant cases. The critical regions in this case are highlighted in red. These interpretations of GRADCAM++ help clinicians validate the model’s outcome and ensure trust in the deploying AI-assisted system for classifying tumors into Malignant and Benign categories.

### 4.1 Comparative analysis

The proposed model’s performance is contrasted with various existing recent techniques designed for Breast Cancer Detection. To improve the robustness and reliability of BC detection systems, researchers have developed various deep learning, machine learning, and multi-modal imaging strategies. This study’s main focus is the analysis of the histopathological images encompassed in the Breast Ultrasound Image Dataset (48), to offer a fair and meaningful comparison as demonstrated in **Table 3**.

**Table 3:** Comparison of proposed methodology with other relevant approaches.

Reference	Model	Accuracy	Precision	Recall	F1_score	XAI technique employed
(59)	VGG19	87.8%	80.8%	83.8%	83.8%	Not Used
(60)	DenseNet	89.87%	91.11%	89.87%	90.00%	GRADCAM
(61)	CNN	96.10%	97.24%	94.12%	95.8%	Not Used
(62)	Deep CNN	90.13%	83.18%	93.54%	88.38%	Not Used
(63)	EfficientNet-B7	91.67%	89.89%	91.95%	90.91%	LIME
(64)	Hybrid Ensemble Model	93.5%	98.4%	98.0%	98.1%	GRADCAM
(65)	XGBoost	85%	85.1%	84.5%	84.7%	SHAP
(40)	Ensemble Learning Model	90%	90%	90%	90%	Not Used
(41)	Voting Learning Model	94.20%	90.63%	96.67%	93.55%	Not Used
(43)	Transfer learning with VGG16	91%	88.75%	94%	91%	GRADCAM
Proposed Approach	Fusion (VGG16+DenseNet121+Xception)	97.14%	95.96%	98.42%	97.18%	GRADCAM++

The use of the VGG19 architecture for classifying BC using ultrasound images is investigated in the article (59). The researchers use transfer learning to make use of the representational power of VGG19. This well-known convolutional neural network has been pre-trained on extensive datasets, later trained on an ultrasound image dataset. A five-fold cross-validation strategy is used during training to reduce overfitting, with an overall classification accuracy of 87.8%. However, the study’s clinical interpretability is limited because explainability processes are not included. An explainable DL framework for BC diagnosis is presented by Alom et al. in (60). This approach uses DenseNet as the central feature extractor along with other convolutional layers and transfer learning. This hybrid architecture aims to extract hierarchical features from ultrasound pictures of the breast. To improve model transparency and trustworthiness—a crucial component of medical AI systems—the researchers employ the Grad-CAM technique. With a classification accuracy of 89.87%, this model exhibits a fair trade-off between interpretability and predictive performance. The authors in (61) present a multi-layer CNN model for binary classification of breast ultrasound pictures into malignant and benign classes. The study maintains high diagnostic performance while emphasizing computational efficiency and architectural simplicity. This CNN model achieves an accuracy of 96.10%. However, this model lacks explainability, which could prevent it from being used in actual clinical operations where interpretability is crucial. The researchers in (62) demonstrate using the deep CNN network with a multi-scale kernel on the breast ultrasound images. This deep network presents a collaborative way of recognizing malignant tumors and detecting the solid nodules among them.

The authors in (63) demonstrate the application of the UNET architecture to segment the breast ultrasound images, and EfficientNetB7 performs the classification. XAI technique LIME is used for explaining the results by outlining the visual representations. This model shows an accuracy of 91%, and the LIME results help the clinicians validate the decisions. The study in (64) proposes an automated DL-based CAD system for diagnosing BC using ultrasound images. This hybrid model combines MobileNetV2, RESNET101, VGG16, and RESNET50. The Grad-CAM technique is used to improve interpretability.

The proposed approach resulted an accuracy of 93.5%. In (65), the authors utilized image-based data to investigate the impact of different ML techniques for BC classification. Among the models studied, the XGBoost approach achieved the most significant classification accuracy of 85%. The study used Shapley Additive Explanations (SHAP), a popular Explainable AI (XAI) technique, to address the interpretability issues frequently associated with ML models. SHAP presents the contribution of various features towards the model’s predictions, revealing significant information about the most influential variables linked with breast cancer diagnosis. The study in (40) presents the meta learning ensemble framework that combines multiple CNN architectures (RESNET50, DenseNet121 and InceptionV3) with data augmentation and transfer learning techniques. This work’s main contribution is optimizing the ensemble output of CNN architectures that have already been trained using a meta-learning approach. This approach recorded the accuracy of 90% when implemented on the Breast Ultrasound Image dataset. This approach uses the explainability technique to interpret the results. In (41), the models AlexNet, ResNet101, and InceptionV3 are used for transfer learning on the ultrasound Image dataset. This approach applies the soft voting technique to present the final prediction based on individual models’ prediction probabilities. The voting learning model recorded the accuracy of 94.20% through a soft-voting ensemble of these networks, with AlexNet (81.16%), RESNET101 (85.51%), while InceptionV3 (91.3%) contributing in that order. Although the study focuses on better model generalization, the dataset size is still a constraint, and explainable AI techniques are not used to evaluate predictions. In (43), the authors used transfer learning with the VGG16 architecture for breast cancer categorization. The model’s performance measures were remarkable, with an accuracy of 91%. To improve model interpretability, the researchers used Grad-CAM to create visual explanations highlighting the key regions in the input photos that influenced categorization results.

The proposed DL fusion model: integrating VGG16, Xception, and DenseNet121 outperforms several cutting edge solutions employed for BC detection using Ultrasound Images with an accuracy of 97.14%. The XAI strategy, which guarantees confidence in the model’s judgments, is not used in a number of the approaches reported in the comparative analysis. In contrast to our approach, several approaches have used XAI techniques, but their accuracy is relatively low. Combining multiple DL architectures enables reliable feature extraction with improved accuracy. Our proposed fusion model extracts the global and local features that result in enhanced performance. The application of GRADCAM++ adds value for physicians and clinicians as they can validate the model’s predictions by seeing the highlighted essential portions of the images dominating the decisions.

## 5 Conclusion

This research proposes a fusion-based DL approach for identifying BC from Ultrasound Images by combining the VGG16, DenseNet121, and Xception models. With a remarkable accuracy of 97.14%, the fused model outperforms the individual models and several current state-of-the-art techniques. Our model successfully extracts low-level structural and high-level semantic characteristics from histopathology pictures by utilizing the complementary capabilities of the various architectures. This work includes using the GRADCAM++ technique to achieve interpretability, which made it possible to present the important sections in the images that lead towards predictions. The model fusion approach results in enhanced accuracy resulting in low false positives and false negatives for Breast Cancer Detection along with explainable results to promote clinical trust which is highly needed in medical setting. In addition to increasing clinical trust, this transparency satisfies the pragmatic requirements of healthcare applications, where it is essential to comprehend the reasoning behind automated predictions.

Apart from the encouraging results, the proposed model faces some difficulties in attaining strong generalization across datasets with different imaging setups and acquisition techniques. Incorporating sophisticated feature selection techniques, improving computational efficiency, and improving generalizability through domain adaptation strategies will be the key goals of future research. We also intend to examine self-supervised and unsupervised learning techniques to further enhance diagnostic accuracy and model resilience in actual clinical situations and employ privacy-preserving techniques to ensure patient data privacy.

## Data Availability

The original contributions presented in the study are included in the article/supplementary material. Further inquiries can be directed to the corresponding author.
